# Evaluation of carp sperm respiration: fluorometry with optochemical oxygen sensor versus polarography

**DOI:** 10.1007/s10695-024-01418-2

**Published:** 2024-12-04

**Authors:** Iryna Musatova, Borys Dzyuba, Serhii Boryshpolets, Azeem Iqbal, Anatolii Sotnikov, Vitaliy Kholodnyy, Viktoriya Dzyuba

**Affiliations:** South Bohemian Research Center of Aquaculture and Biodiversity of Hydrocenoses, Faculty of Fisheries and Protection of Waters, University of South Bohemia in České Budějovice, Zátiší 728/II, 389 25 Vodňany, Czech Republic

**Keywords:** Common carp, Spermatozoa, Oxygen consumption rate, Clark-type electrode, Optochemical oxygen sensor, Sperm motility

## Abstract

The primary function of spermatozoa is to fertilize the oocyte, which depends on their motility and is directly associated with their metabolic state. The oxygen consumption rate (OCR) of spermatozoa reflects the respiratory capacity of sperm mitochondria under various physiological conditions and is an essential marker of sperm quality. We determined the OCR of common carp (*Cyprinus carpio*) sperm using two respirometry methods: the conventionally used polarographic method with a Clark-type electrode and fluorometric assay with an Oxo Dish optochemical oxygen sensor. The latter was used for the first time to evaluate spermatozoa oxygen consumption in various metabolic states (under different treatments) at different dilution rates. These two methods were compared using Bland–Altman analysis, and the applicability of the optochemical oxygen sensor for evaluating carp sperm oxygen consumption was discussed. Sperm motility and progressive velocity parameters were also assessed to evaluate the effect of sperm respiration under different metabolic states and dilution rates and preincubation period on the physiological status of spermatozoa. The comparison of these respirometry methods clearly shows that while the polarographic method allows immediate measurement of oxygen levels after adding a sperm sample, the optochemical oxygen sensor has a priority in the amount of data obtained due to simultaneous measurements of several samples (e.g., different males, different fish species, repetitions of the same sample or various experimental conditions), even at a later time after adding sperm to the measuring chamber. However, the compared methods are complementary, and the proposed methodology can be applied to other fish species.

## Introduction

The primary function of sperm is to activate motility, reach the egg, and fertilize it—these processes require energy obtained through various biochemical pathways. In contrast to mammals or other internal fertilizers, sperm in most externally fertilizing fish species activate their motility only after contact with the environment for quite a short time (1–2 min in freshwater fishes and up to tens of minutes in some marine fishes). The energy supply pathways before and during this activation may vary across fish species and depend on the physiological state of the spermatozoa (motile or immotile) (Dzyuba et al. 2017). Quiescent fish spermatozoa utilize oxygen for oxidative phosphorylation, essential for maintaining ion exchange across the plasma membrane and generating ATP reserves, crucial for initiation of spermatozoon motility, but insufficient to support motility for a long (or relatively long) time (Rahi et al. 2021). This is a possible reason why actinopterygian fish sperm use different energy pathways for supporting motility, including glycolysis, oxidative phosphorylation, tricarboxylic acid cycle, lipid anabolism and catabolism (Cosson 2019; Mansour et al. 2003). Species-specific differences in the utilization of various metabolic substrates for spermatozoa ATP production (Dzyuba et al. 2017; Mansour et al. 2003) and in the efficiency of enzymatic systems involved in energy supply for flagella motility were reported (Dzyuba et al. 2016; Gronczewska et al. 2019). Analysis of the sturgeon sperm proteome showed that the bulk of proteins activated in high-quality sperm are associated with redox reactions, “generation of metabolite and energy precursors” and are localized in mitochondria (Dietrich et al. 2019). Active mitochondrial oxygen consumption and oxidative phosphorylation were shown in both immotile and motile Siberian sturgeon spermatozoa (Rahi et al. 2021). It is believed that there is no correlation between basal sperm respiration rate in a quiescent state and sperm motility after activation. However, sperm motility was shown to correlate with accumulated ATP content in fish spermatozoa (Ingermann et al. 2003; Perchec et al. 1995). The functioning of sperm mitochondria and ATP production during respiration may also be associated with other important tasks: producing reactive oxygen species (ROS), regulating intracellular Ca^2+^ levels, and signaling. Studying fish sperm respiration can provide valuable information on mitochondrial function, metabolism, and its dysregulation, which is essential for understanding sperm bioenergetics and assessing sperm quality and fertilizing potential. However, comprehensive, reliable, and convenient methods are highly required to address the issue. Various methods exist to measure oxygen consumption (Wang and Wolfbeis 2014), differing in their applicability, data acquisition rate, accuracy, required sample volume, ease of operation, and costs. Popular methods in biology and medicine include electron paramagnetic resonance, polarography, and fluorometry.

Polarography, a conventional method, is widely used to measure oxygen consumption in isolated mitochondria and whole cells. The measurement of oxygen consumption using a Clark-type electrode is based on the electrochemical reaction of oxygen reduction on the platinum electrode (Chang et al. 1993; Park et al. 1986; Zhang and Anderson 2007). While the polarographic method enables immediate respiration measurement after introducing a biological object and various agents into the measuring chamber, it has limitations when examining many samples simultaneously. Other devices, including those with optochemical oxygen sensors, have been developed to address these limitations. The optochemical sensor operates on the principle of fluorescence quenching in the presence of dissolved oxygen using oxygen-sensitive dyes (luminophore). The fluorescence quenching is measured by detecting the difference between the fluorescent and reference signals and transforming them into an oxygen concentration (Gouspillou et al. 2011; Schuh et al. 2011). However, in method comparison studies, the statistical approach to assessing the degree of agreement is not apparent, the choice of a correct statistical approach to assessing the degree of agreement is not obvious, and the error of the methods being compared is of great importance (Magari 2004). Commonly used correlation and regression analyses evaluate the relationship between variables, not the differences. That is why it is not optimal and should not be recommended for assessing the comparability of methods.

Our study aimed to compare fluorometric and polarographic methods for measuring carp sperm oxygen consumption. We choose a Bland and Altman proposed analysis method based on the quantification of the agreement between two measurements by identifying the mean difference and limits of agreement and constructing a plot for estimation of an agreement interval, within which fall 95% of the differences of the second method compared to the first one (Bland and Altman 1986; 2003). Considering that sperm oxygen consumption is mainly related to mitochondrial respiration (Moraes and Meyers 2018) and that mitochondrial oxidative phosphorylation is dependent on oxygen concentration in the medium (Wilson et al. 1979), different sperm dilution rates were used to test the effect of oxygen concentration on sperm respiration. Since the motility is implemented by using sperm ATP previously accumulated mainly due to oxidative phosphorylation (Figueroa et al. 2017; Rahi et al. 2021), we tested the effect of oxygen consumption level in immotile sperm on sperm performance (motility and velocity characteristics) during following motility. The obtained results provide insights into the applicability and limitations of the each used method, contributing to the advancement of fish reproduction studies.

## Materials and methods

### Fish maintenance and sperm sample collection

The study was conducted following the principles of the Ethics Committee for the Protection of Animals in Research of the University of South Bohemia in Ceske Budejovice, Research Institute of Fish Culture and Hydrobiology, Vodnany, based on the EU-harmonized Animal Welfare Act of the Czech Republic. Manipulations with animals were performed by the authorization for the use of experimental animals (reference number: 68668/2020 MZE-18134 and 68763/2020-MZE-18134) and with the authorization for breeding and delivery of experimental animals (reference number: 64155/2020-MZE-18134) issued to the Faculty of Fisheries and Protection of Waters, University of South Bohemia, by the Ministry of Agriculture of the Czech Republic. In the experiments, mature males of common carp (*Cyprinus carpio*) kept at the Genetic Fisheries Centre, Faculty of Fisheries and Protection of Waters, Vodnany, Czech Republic, were used. Spermiation was induced by intramuscular injection of carp pituitary powder (1 mg/kg bw) dissolved in 0.9% NaCl solution 24 h before sperm collection. Semen samples were collected by abdominal massage into 10-mL syringes and kept on ice until used in the experiments. Samples with sperm motility higher than 80% were used in the study.

### Working solutions

Non-activating medium (NAM) contained 100 mM NaCl, 40 mM KCl, 2 mM CaCl_2_, 1 mM MgSO_4_, and 20 mM Tris (pH 7.5; 298 mOsm/kg), and activating medium (AM) had 100 mM NaCl and 10 mM Tris (pH 9.0; 195 mOsm/kg) (Cejko et al. 2022). Potassium cyanide (KCN) inhibitor and carbonyl cyanide 3-chlorophenylhydrazone (CCCP) uncoupler of oxidative phosphorylation were used with the final concentrations of 1 mM and 2 μM, respectively (concentrations were estimated during preliminary trials).

### Experimental design

A comparison of Clark-type polarographic oxygen electrode (Polarograph) and SDR Sensor Dish® Reader (SDR) was undertaken to assess the applicability of the optochemical method for measuring oxygen consumption by fish sperm, in particular, determination of the oxygen consumption rate (OCR) of spermatozoa in a non-activated or activated state, with an uncoupler or inhibitor of oxidative phosphorylation, at different sperm dilution rates. Since the level of oxygen consumption may change during measurement (differently in different physiological states and with different additives), OCR assessments were performed at the beginning, middle, and end of the incubation period of 17 min. The physiological state of spermatozoa during experiments was also characterized by examining their kinematic parameters and motility percentage.

The experimental procedure comprised the following stages:

1. Data on the oxygen content in the measuring chamber during sperm incubation in NAM at dilution rates of 10, 25, and 50 (the ratio of carp sperm to NAM is 1:9, 1:24, and 1:49, respectively) using a Polarograph and SDR were obtained for the evaluation of sperm oxygen consumption simultaneously with estimation of its dependence on sperm dilution rate.
2. Bland and Altman’s analysis was used to compare the Polarograph and SDR using obtained oxygen content data.
3. The OCRs from oxygen content data were calculated for SDR at selected dilution rates in AM or NAM with an inhibitor or uncoupler of oxidative phosphorylation.
4. Spermatozoa motility, straight-line velocity, and linearity were estimated after incubation in NAM for 860 s (with and without uncoupler or inhibitor of oxidative phosphorylation at different sperm dilution rates to correspond to the conditions during oxygen consumption estimation). Motility was initiated in the AM with and without the same additives as during preincubation.

### Clark electrode-based measurement of oxygen content

A Clark-type polarographic oxygen electrode (YSI 5300A Biological Oxygen Monitor; OH, USA) was used to determine the oxygen content in a measurement chamber with a water jacket and magnetic stirrer (IKA magnetic stirrer; Staufen, Germany). The air-saturated media were added into the device chamber and incubated with stirring at 800 rpm until a steady baseline was obtained. The water circulation system connected to the thermostat maintained a stable temperature (20 °C) in the chamber. A sperm sample of the needed volume (depending on the required sperm dilution rate) was added to the device chamber with NAM or AM, and endogenous respiration, respiration inhibited by KCN, and uncoupled respiration by adding CCCP were recorded. The oxygen content in the chamber was measured in real-time for 17 min with an interval of 1 s and expressed as µmol O_2_/10^9^ spermatozoa (spz).

### Fluorescence-based measurement of oxygen content

The oxygen content was estimated using the integrated Oxo Dish optical-chemical oxygen sensor of SDR Sensor Dish® Reader (Precision Sensing GmbH, Germany) and instrument software following the instructions provided by the manufacturer. For fluorescence-based measurement of oxygen content, 340-nm excitation and 642-nm emission filters were used. Sperm samples of the needed volume (depending on the required sperm dilution rate) were added to a 24-well plate of the measuring chamber, placed in the SDR, maintained at a temperature of 20 °C (by the water circulation system connected to the thermostat), and constantly shaken on a rocking shaker (IKA Rocker 2D basic, China). Oxygen content values in the plate were recorded every 15 s for 17 min using Micro Resp® version 1.0.4 (Loligo Systems, Viborg, Denmark) software. The oxygen content values obtained were corrected against the blank (basic slopes in the absence of cells), normalized against the initial intensity (oxygen content in the measuring chamber with NAM at 20 °C), and expressed as µmol O_2_/10^9^ spz.

### Calculation of sperm oxygen consumption rate

Obtained data were analyzed by regression analysis according to McDonald (2014), which revealed the appropriateness of the application of polynomial regression of the second order for the description of the time dependency of oxygen content in the chamber when *r*^2^ > 0.99, which was statistically higher than *r*^2^ for linear regression. Further increasing of polynomial degree resulted in negligible *r*^2^ raise. Thus, sperm OCR was calculated as the derivative of the resulting polynomial function concerning time and considering sperm concentration in the measuring chamber and was expressed as (nmol O_2_/min) per 10^9^ spz. The rate of uncoupling of oxidative phosphorylation in carp sperm was calculated as the ratio of the OCR of sperm in the uncoupled state (in NAM or AM with the addition of CCCP) to the OCR in the coupled state (in NAM or AM without the addition of CCCP).

### Spermatozoa motility and sperm concentration evaluation

Motility of fresh sperm samples and spermatozoa after preliminary incubation in NAM with or without KCN or CCCP was recorded 60 s post-activation in AM using the ISAS sperm track-10 sperm counting chamber (PROISER, Spain), a phase contrast microscope with 10 × magnification (PROISER, Spain), and an IDS digital camera (IDS Imaging Development Systems GmbH, Germany). The video records were analyzed using the CASA plugin for ImageJ (Purchase and Earle 2012; Wilson-Leedy and Ingermann 2007). Percentage of motile spermatozoa (%), curvilinear velocity (VCL, μm/s), average path velocity (VAP, μm/s), straight-line velocity (VSL, μm/s), linearity of the path (LIN, straight-line velocity/average path velocity), oscillation of the track (WOB, average path velocity/curvilinear velocity), and beat-cross frequency (BCF, Hz) were estimated. Only VSL and LIN were used to present the results (see “Statistical analyses” for details).

Sperm concentration in the samples was evaluated using a Burker cell hemocytometer (Meopta, Czech Republic) and Olympus BX 50 phase contrast microscope (200 × magnification; Olympus, Japan).

### Statistical analyses

A comparison between devices was made by assessing the repeatability of the data obtained according to Bland J.M. and Altman D.G. (Bland and Altman 1986; 2003). For this, the differences between values obtained by the first (Polarograph) and the second (SDR) methods versus the average between the values obtained by both methods were plotted, and the 95% limits of agreement (LOA) were calculated for paired observations.

Because sperm OCR and uncoupling rate were changed with time after sperm dilution, data obtained from measuring sperm respiration using the optochemical method were analyzed at three time points after sperm dilution: 320, 590, and 860 s. As the data were not normally distributed in all experimental groups (*p* < 0.05, Shapiro–Wilk test), a non-parametric variance analysis was performed using the Kruskal–Wallis test followed by multiple comparisons of mean ranks for all groups. This approach was applied to compare the effects of time after dilution and dilution rate separately. Additionally, comparisons between OCR and uncoupling rate in AM versus NAM were performed using the Mann–Whitney test. Statistical hypotheses in the applied tests were rejected at *p* < 0.05.

The data set of kinetic parameters obtained by CASA (altogether the tracks of 1.94 × 10^6^ spermatozoa were obtained) were analyzed using Spearman’s rank test. VSL and LIN as parameters with a lower correlation coefficient (*r* < 0.11) were selected to present the results. Further, the obtained individual spermatozoon VSL and LIN data were averaged for each male at each experimental condition and post-activation time. The averaged by male VSL, LIN, and motility percentage were used to plot the lines reflecting the changes of these parameters over the motility period.

Statistical data analysis was conducted using Statistica (version 14, TIBCO Software Inc., 2020, Palo Alto, CA, USA), RStudio® (Posit Software, PBC), and Microsoft Excel.

## Results

### Changes in oxygen consumption in NAM at different sperm dilution rates

**Fig. 1:**
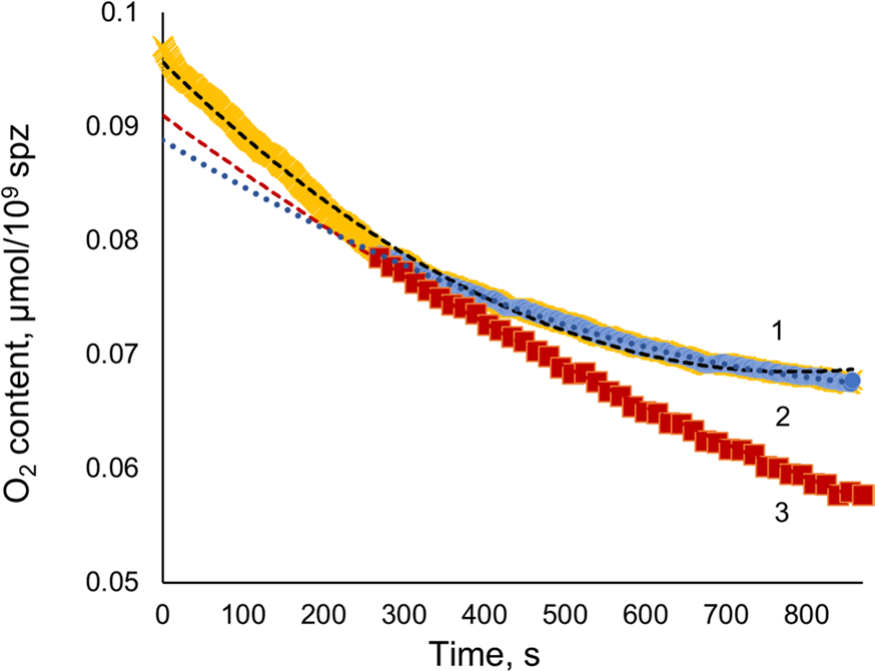
Examples of the curves of O_2_ content changes over time at sperm dilution rate 10, registered by two methods: polarographic—yellow line 1 (0–270 s) + blue line 2 (270–870 s), optochemical—red line 3 (270–870 s). The approximation by polynomial regression lines of the 2nd order is indicated by corresponding dotted lines: (1) y = 4∙10^−8^x^2^ − 7∙10^−05^x + 0.0957, *R*^2^ = 0.994; (2) y = 2∙10^−08^x^2^ − 4∙10^−05^x + 0.0888, *R*^2^ = 0.998; (3) y = 10^−08^x^2^ − 5∙10^−05^x + 0.091, *R*.^2^ = 0.999

The oxygen consumption curves for carp sperm were built based on the data for oxygen content in the measuring chamber within 17 min using Polarograph and SDR after adding carp sperm to the measuring chamber (Fig. 1).

Due to the time needed to add sperm samples to SDR’s measuring chambers and assemble the reader, the recording was started with a delay of several minutes (270 s in the example shown). In contrast, in the case of the Polarograph, the recording was started immediately after adding sperm samples. Therefore, for comparative analysis, only a particular part of the data (corresponding to 270–870 s after sperm addition) was used. The data recorded by both devices every 15 s at the same time after sperm addition into the measuring chamber was taken for further processing.

We checked if it would be possible to approximate the oxygen content line (270–870 s), recorded by the optochemical method, to obtain a line of oxygen content for the initial period of sperm respiration (0–270 s) lost for the abovementioned technical reasons. The approximation of the 0–270 s interval (using the 2nd order polynomial regression and experimental data for 270–870 s interval) for both Polarograph and SDR data sets and comparison of the approximated curve with experimental one obtained by Polarograph clearly showed the invalidity of such approach.

### Comparison of Polarograph and SDR oxygen consumption data for carp sperm according to Bland and Altman

**Fig. 2:**
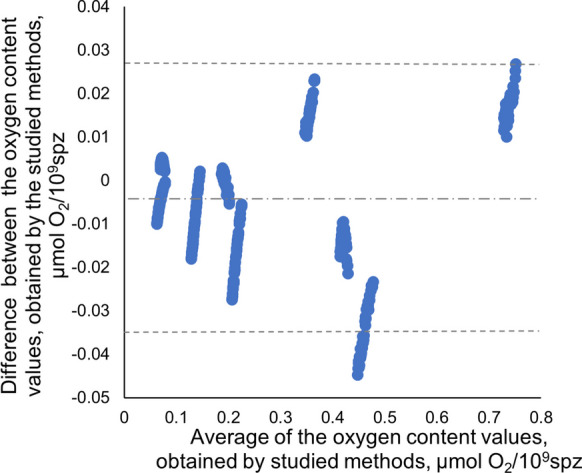
Bland and Altman plot for O_2_ content data, obtained by Polarograph and SDR. The dotted lines delimit the LOA area (mean ± 1.96 SD); the dash-dot line indicates the mean of difference

According to Bland and Altman (1986), formal analysis tends to give too large LOA, which can lead to the adoption of inadequate measurement methods. That is why estimating the LOA (the interval within which 95% of the measurement differences are expected to lie) is necessary. The two measurement methods can be used interchangeably if differences within this interval are not significant (Pum 2019). The results of the LOA assessment showed that the differences within mean ± 1.96 SD (Fig. 2) were greater at the highest dilution rates (≥ 50), corresponding to carp sperm concentration ≤ 1.223 × 10^9^ spz/mL. Differences in the O_2_ content obtained by the studied methods for lower dilution rates of the sperm samples (10 and 25) were within mean ± 1.96 SD. Thus, no significant differences are expected between the data obtained by the two compared methods when a higher number of cells are in the measuring chamber. So, for concentrated sperm samples, the two measurement methods can be used interchangeably.

### Calculation of sperm OCRs from SDR oxygen content data at different sperm dilution rates and metabolic states

Given the interchangeability of the two studied methods for measuring sperm respiration, identified by the Bland and Altman method, SDR was used to obtain data on sperm respiration, and sperm oxygen consumption rates were calculated at different dilution rates, activated and non-activated metabolic states, and at different time points after sperm dilution.

**Fig. 3:**
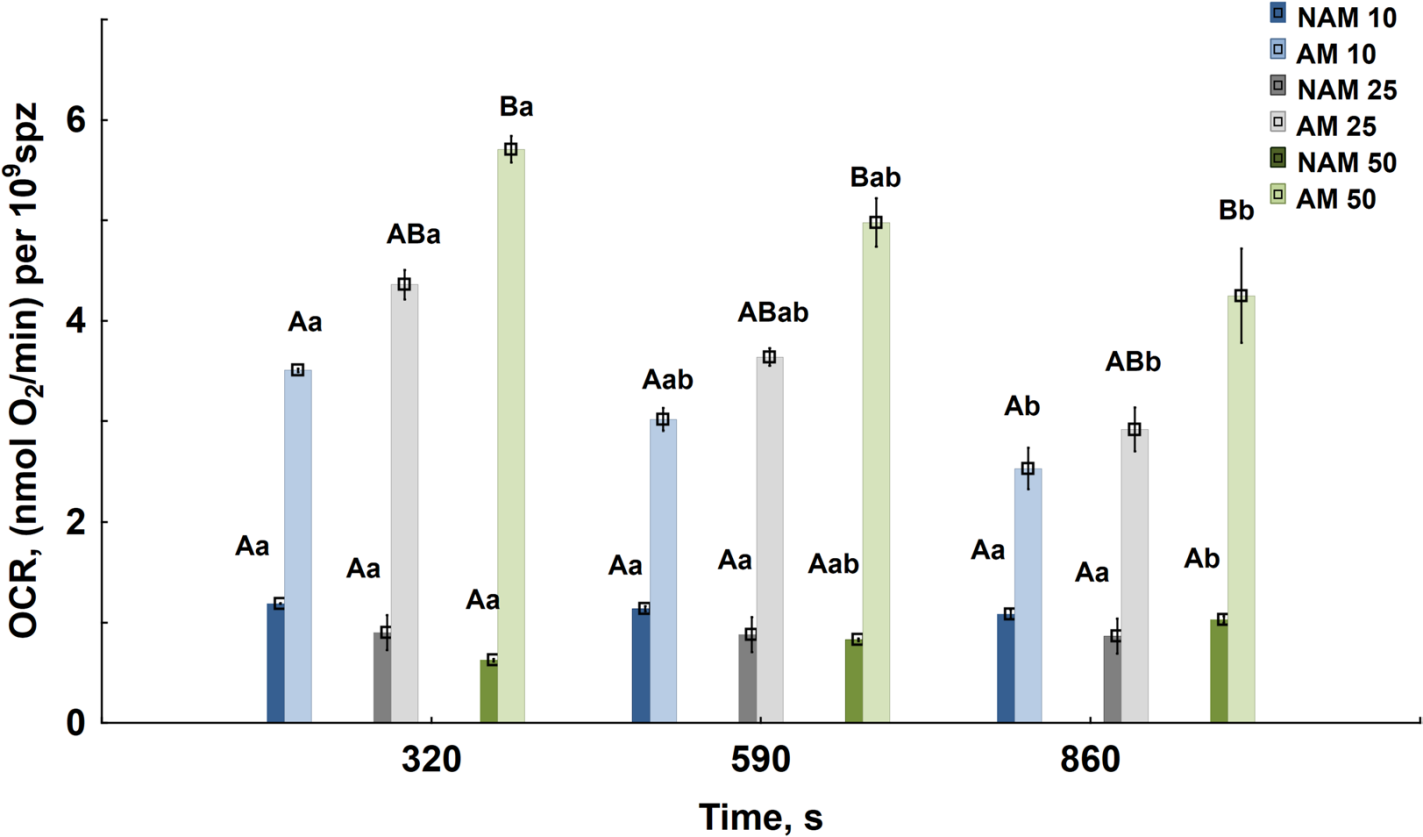
Oxygen consumption rate (OCR) of carp sperm in non-activated (NAM) and activated (AM) metabolic states at different time points after sperm dilution. NAM 10, NAM 25, NAM 50 and AM 10, AM 25, AM 50 corresponds to OCR in NAM or AM at sperm dilution rates 10, 25, and 50, respectively. Different capital letters indicate significant differences among the values in groups with different dilution rates or in different metabolic states at the same time point. Different lowercase letters indicate significant differences among the values in groups at the same dilution rate and metabolic state within the different time points. The means of groups marked with the same letter are not significantly different (Kruskal–Wallis’s test). Kruskal–Wallis’s test, followed by multiple comparisons of mean ranks for all groups, *p* < 0.05. At each dilution rate and time point, OCR in AM is different from OCR in NAM (Mann–Whitney’s test, *p* < 0.05)

In activating conditions, the OCR of spermatozoa was found to be significantly decreased over time and significantly increased with an increase in the degree of dilution from 10 to 50, in contrast to NAM, in which sperm OCR was significantly lower than in AM and did not differ between sperm dilutions at the same time point (Fig. 3). Thus, the quantity of consumed O_2_ and, respectively, sperm OCR depends on each of the studied factors.

The pattern of the dependence of sperm OCR in AM on the dilution rate does not change over time. It should be noted that the intensity of sperm respiration during the observation period was significantly higher in AM than in NAM. In AM, OCR increased with the dilution rate and became significantly higher at a dilution rate of 50 compared to a dilution rate of 10.

**Fig. 4:**
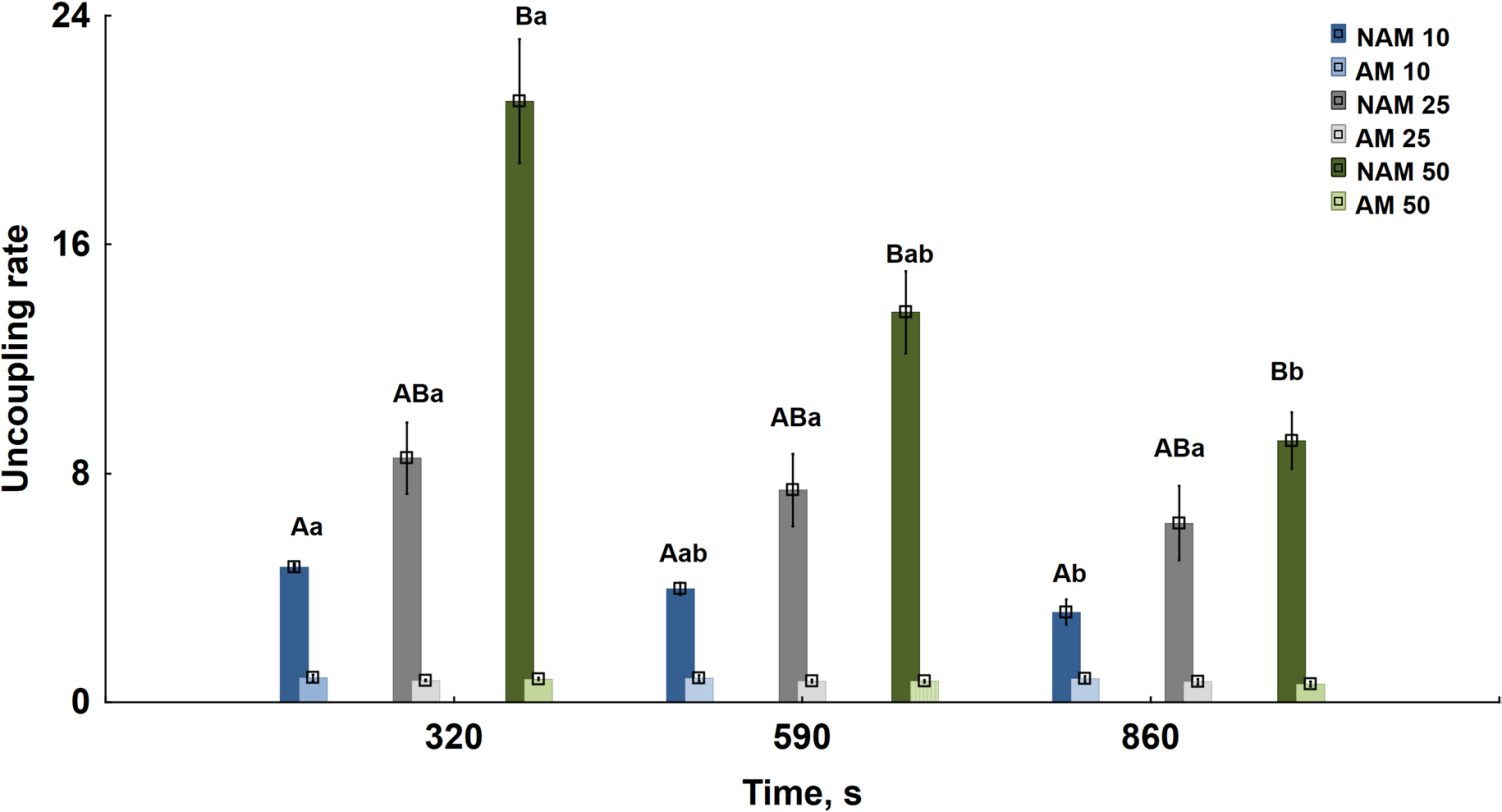
Uncoupling rates of carp sperm oxidative phosphorylation in non-activated (NAM) and activated (AM) metabolic states at different time points after sperm dilution. NAM 10, NAM 25, NAM 50 and AM 10, AM 25, AM 50 corresponds to uncoupling rates in NAM or AM at sperm dilution rates 10, 25, and 50, respectively. Different capital letters indicate significant differences among the values in groups with different dilution rates or in different metabolic states at the same time point. Different lowercase letters indicate significant differences among the values in groups at the same dilution rate and metabolic state within the different time points. The means of groups marked with the same letter are not significantly different (Kruskal–Wallis’s test). Kruskal–Wallis’s test, followed by multiple comparisons of mean ranks for all groups, *p* < 0.05. At each dilution rate and time point, uncoupling rates in AM are different from uncoupling rates in NAM (Mann–Whitney’s test, *p* < 0.05) and do not differ significantly between groups in NAM with the same dilution rate and at different time points (Kruskal–Wallis’s test)

At the same time, a significant and even more pronounced dependence of sperm OCR on dilution rate was observed in NAM upon uncoupling sperm oxidative phosphorylation by adding CCCP (Fig. 4). There was an increase in sperm uncoupling rate at dilution rate 50 compared to 10 and a significant decrease in this value at the end of the observation period. However, there was no difference in the uncoupling rate at dilution rate 25 compared to 50 and 10 dilution rates, and there was no decrease in the uncoupling rate over time at this rate of sperm dilution. It turned out that the rate of uncoupling of oxidative phosphorylation of carp spermatozoa in the non-activated state is significantly higher than in the activated state at the studied sperm dilution rates and time points. Adding KCN reduced respiration to fluctuating near-zero OCR values in both activated and non-activated states (these results were not included in the statistical analysis).

### Assessment of carp sperm motility parameters after preincubation in NAM at different conditions

**Fig. 5:**
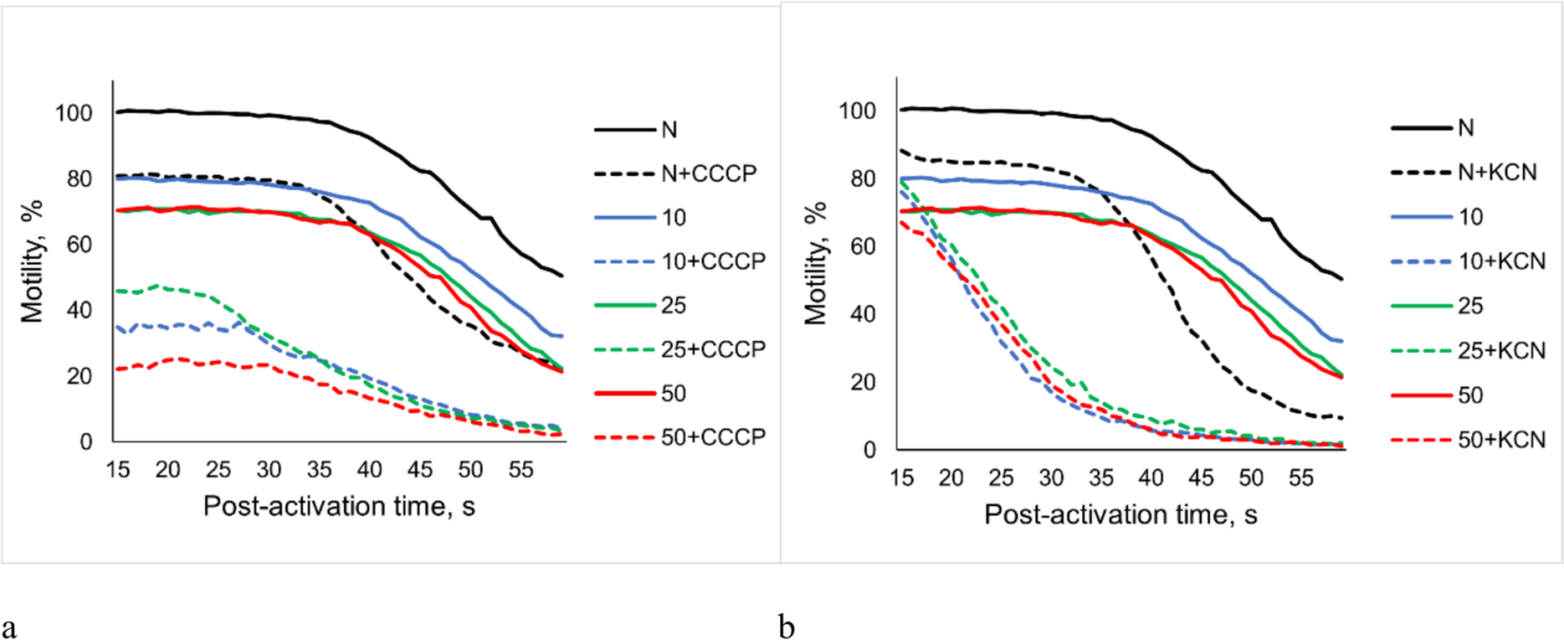
Carp sperm motility percentage in AM after preincubation in NAM with an uncoupler CCCP (**a**) or inhibitor KCN (**b**) of oxidative phosphorylation. Undiluted sperm (N) without preincubation—black lines. Colored lines correspond dilution rates: blue—in 10, green—in 25, and red—in 50 times. Solid lines—control (spermatozoa without additives), dotted lines—with addition of an uncoupler (2 μM CCCP) or inhibitor (1 mM KCN)

The percentage of carp sperm motility after preincubation in NAM for 860 s at all studied sperm dilution rates decreased over post-activation time in AM. It was lower than the motility of undiluted non-preincubated sperm (Fig. 5).

There was a similarity in the characteristic pattern of changes in the motility of (1) undiluted spermatozoa without preincubation, (2) diluted preincubated with CCCP or KCN, and (3) spermatozoa preincubated with the addition of CCCP at all dilution rates studied: sperm motility was stable for 35–40 s, and then slowly decreased. However, the motility percentage of all diluted sperm samples preincubated in NAM containing KCN decreased sharply from 15 s post-activation. In comparison, the motility percentage of sperm without dilution and preincubation in the presence of KCN remained constant up to 30 s post-activation (Fig. 5b).

**Fig. 6:**
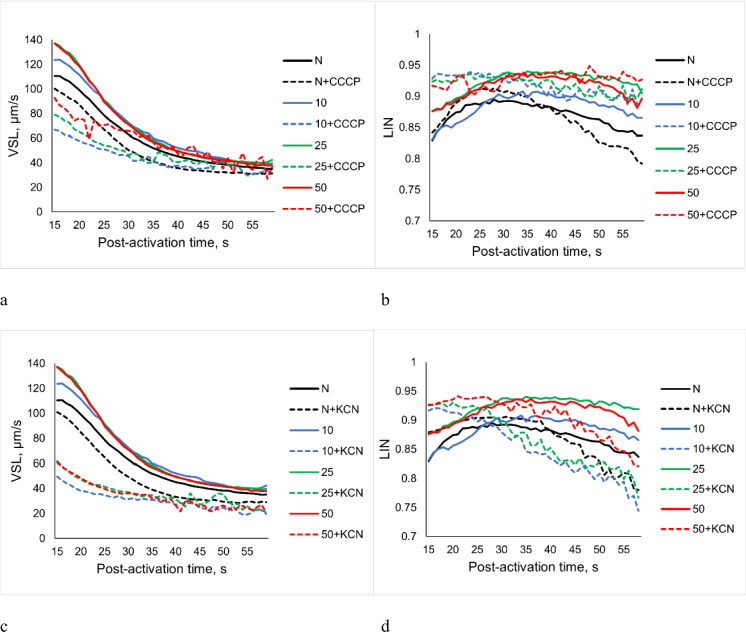
Straight-line velocity (**a**, **c**) and linearity (**b**, **d**) of carp spermatozoa in AM after preincubation in NAM with an uncoupler CCCP (**a**, **b**) or inhibitor KCN (**c**, **d**) of oxidative phosphorylation. Undiluted sperm (N) without preincubation—black lines. Colored lines correspond dilution rates: blue—in 10, green—in 25, and red—in 50 times. Solid lines—control (spermatozoa without additives), dotted lines—with addition of an uncoupler (2 μM CCCP) or inhibitor (1 mM KCN)

Preincubation of diluted sperm led to a dilution rate-dependent increase of VSL values compared with undiluted sperm, which was especially noticeable within 30 s post-activation (Fig. 6a). Activation of diluted sperm preincubated in NAM with the addition of CCCP caused an essential decrease in VSL compared to diluted preincubated sperm without the addition of uncoupler and was higher, the higher the dilution rate (Fig. 6a). At the same time, 15 s after activation, a pronounced increase in LIN was found for diluted sperm preincubated with CCCP, which remained higher than for non-preincubated with and without CCCP undiluted sperm throughout the observation period (Fig. 6b).

After preincubation of sperm at the studied dilution rates in NAM with the addition of KCN at the beginning of the observation period and during 25 s, the VSL values were lower, and the LIN values were higher than the corresponding parameters of preincubated diluted sperm without the addition of KCN (Fig. 6c, d). LIN values of diluted sperm treated with KCN decreased over time and reached values lower than those of undiluted non-preincubated sperm with the addition of inhibitor, except for sperm in dilution rate 50. It should be noted that the decrease in the parameters of sperm progressive motility (VSL and LIN) after preincubation with a respiration inhibitor was more pronounced compared with the same parameters of sperm preincubated with an uncoupler.

## Discussion

The Bland–Altman procedure and difference plots for comparing the two methods suggested that tested device (SDR) can be used to measure the oxygen consumption of carp sperm along with the conventional device (Polarograph), if the sperm dilution rate is below 50. Nearly 5% of the values of differences do not fall within the 95% LOA and significantly exceed them. That is evidence of a good agreement between the two methods studied. Unlike a Polarograph, which allows continuous recording of oxygen consumption throughout the experiment but only for one sample, the SDR Sensor Dish® Reader allows simultaneous determination of the oxygen content in 24 samples in wells of a touch plate. One computer can control up to 10 touch plate readers with 240 samples for extensive parallel testing. In contrast to polarographic oxygen electrodes, optical oxygen sensors have a faster response time due to fast light signal transduction, less signal drift, higher stability against electrostatic noise, and superior resolution of the slow transitions in oxygen consumption rate (Van Bergen et al. 2014). However, due to the time required to add samples into multiple measurement chambers, it is impossible to get SDR readings and data on the oxygen consumption immediately after sample addition. If this is crucial for the study design, the polarographic method should be used. At the same time, using SDR, we can study the respiration of spermatozoa in various physiological states and under different treatments, even at a later period after adding sperm to the measuring chamber. The undeniable advantage of SDR is the simultaneous acquisition of a sufficient statistical volume of data on oxygen consumption to compare semen samples from different males, different fish species, or sperm in different metabolic states, significantly reducing the duration of the experiment. Thus, the SDR Sensor Dish® Reader expands the possibilities of studying sperm physiology and mitochondrial respiratory activity.

The respiration rate of sperm suspension during the experiment depends on several factors, such as time after sperm dilution, spermatozoa concentration in the sample, physiological state of sperm, and fish species. Changes in sperm respiration in the presence of uncouplers or inhibitors of oxidative phosphorylation are well known (Boryshpolets et al. 2009a; Cosson 2019; Rahi et al. 2020), as well as species-dependent differences in the oxidative metabolism of fish sperm (Ingermann et al. 2003; Rahi et al. 2021). However, according to our research, the first two points require attention when creating the right conditions for measuring sperm respiration.

Our study showed that the OCR of carp sperm in the non-activated state corresponds to already documented values (Boryshpolets et al. 2009a; Ingermann 2008). Using SDR data on oxygen consumption by carp sperm, a significant dependence of sperm OCR on the factors “dilution rate,” “metabolic state,” and “time point” was discovered. Significant stimulation of OCR of sperm in the activated state with an increase in the degree of sperm dilution to 50, not associated with a dilution-related decrease in osmolarity, may be a consequence of the specific respiratory activity of sperm when the amount of oxygen and respectively ROS per sperm cell increases significantly with the rate of dilution. However, no significant differences were observed between the OCR values of sperm groups at different dilution rates in the non-activated state. These results should be considered when using SDR to measure oxygen consumption in spermatozoa with low cell concentrations, and further research is required.

In carp, a significant increase in sperm OCR was observed in the uncoupled state in NAM, which decreases over time and makes it possible to estimate the rate of uncoupling and, indirectly, the maximum metabolic ability of sperm mitochondria to produce ATP at the peak of respiratory activity in the non-activated state. The relationship between the rate of uncoupling and the rate of sperm dilution persisted over time. The lack of significant differences between the data obtained by the SDR on OCR of spermatozoa activated by AM with and without CCCP did not allow the use of AM to assess the respiration of carp spermatozoa in an uncoupled state.

This is probably due to the time delay in adding sperm samples into the SDR device’s series of measuring chambers. High OCR of carp sperm in the first 3 min after incubation in AM + CCCP and a rapid decrease in respiratory capacity occurred, so there is no observed significant difference between OCR in AM and OCR in AM + CCCP in the later incubation period. Potassium cyanide addition significantly reduced sperm respiration compared to the respiration level without KCN in all experimental conditions.

Many studies have shown species-dependent differences in fish sperm respiration in different metabolic states and supplements. As noted (Rahi et al. 2021), the burbot sperm OCRs in AM and NAM differed insignificantly and were not enhanced after uncoupler treatment. In contrast, the OCR of sturgeon spermatozoa in AM was significantly higher than in NAM and was increased in NAM upon uncoupler addition (Rahi et al. 2020). Boryshpolets et al. observed a significant and temperature-dependent increase in carp sperm respiration in an activated state (Boryshpolets et al. 2009a). Such differences in the respiratory activity of sperm are apparently associated with differences in the bioenergetics of freshwater fish that live and spawn in water with different habitat conditions, e.g., temperature, which may influence the reproductive strategy of freshwater species and should be considered.

It is known that sperm dilution in a proper medium is the main factor in the induction of sperm motility. The effect of enhancing sperm motility with increased sperm dilution rate was observed in *Acipenser persicus* after activation in fresh water and buffered fresh water (Alavi et al. 2004). It is believed that during the motility period, fish sperm use the ATP, produced in an immotile state prior to activation, and in common carp, a 2.5–fivefold reduction of ATP reserve after the motility period was observed (Boryshpolets et al. 2009b; Perchec et al. 1995). We assumed that changes in the bioenergetic status of diluted sperm in a non-activated state, preincubated with different additives, can affect sperm respiratory activity, motility, and velocity parameters.

According to our study, the preincubation of spermatozoa in NAM during the 860 s results in sperm OCR increasing with an increasing dilution rate compared with the OCR of non-preincubated spermatozoa. It was found that in mammals, an increase in oxygen consumption by sperm is associated with mitochondrial function and, at the same time, positively correlates with parameters of viability, motility, and velocity of sperm (Darr et al. 2016), as motility requires ATP production by sperm mitochondria (Darr et al. 2017; Plaza Davila et al. 2015). The increase in OCR with increasing sperm dilution may manifest as increased sperm aerobic metabolic plasticity, which appears to maximize the chances of sperm meeting eggs and has been confirmed for 49 species, ranging from protostomes to humans (Potter et al. 2024).

After activation in the AM, sperm motility decreases, and the VSL and LIN of diluted preincubated sperm increase significantly compared to undiluted sperm without preincubation. Over time, the VSL decreases, but the LIN changes slightly, maintaining high values, which agrees with the results, obtained by other authors, who observed a decrease in velocity and motility percentage, but not LIN, relative to time (Wilson-Leedy and Ingermann 2007). Thus, as the dilution rate increases and the overall motility percentage decreases, a portion of the preincubated sperm retains higher VSL and LIN. In some mammalian sperm, a proportion of sperm with hyperactive motility was observed, with a simultaneous decrease in ATP concentration and sperm motility percentage during storage at 18 °C. The CASA variable, LIN, significantly affected animal productivity (Tremoen et al. 2018). The response of sperm motility parameters to storage was explained by a binary pattern: in diluted sperm, the quantitative indicator (percentage of motile spermatozoa) decreases, but the qualitative indicator (percentage of spermatozoa with fast linear movement) is maintained regardless of the duration of storage (Henning et al. 2014). Progressive motility parameters are considered favorable for fertilization, as has been shown for human sperm (Mendizabal-Ruiz et al. 2022). The sperm velocity of one male relative to the other male was shown to be the best predictor of male sperm competition and fertilization success in Arctic charr (Liljedal et al. 2008) and Atlantic salmon (Gage et al. 2004). These studies have shown a mechanism that allows some spermatozoa to maintain progressive motility for successful fertilization.

After preincubation in NAM with the addition of CCCP for 860 s, sperm OCR and rate of uncoupling increased significantly depending on the sperm dilution rate. At the same time, sperm motility after preincubation in specific conditions and further activation decreased in a dilution-dependent manner. Thus, the uncoupler causes an increase in respiration during preincubation, affecting the ability of sperm to move after activation. The effects of CCCP, associated with dissipation of the mitochondrial proton gradient, depletion of cellular ATP (Zhang et al. 2021), and interaction with the sulfhydryl groups of dynein ATPases (Hollenbeck et al. 1985), may be reasons for the decrease in motility and velocity parameters after preincubation of carp sperm with CCCP. The longer the time of sperm incubation, the more pronounced the reduction in motility will be obtained. The dependence of the rate of uncoupling on the rate of dilution during the preincubation period indicates an increase in the ability of spermatozoa to consume oxygen as sperm concentration decreases.

While the OCR of sperm in NAM supplemented with KCN during incubation was minimal, preincubation of diluted sperm in NAM with the addition of KCN does not significantly decrease their motility at the initial period after activation compared to the motility of non-preincubated spermatozoa with the addition of an inhibitor or preincubated diluted sperm. A reasonable explanation for the relatively high motility after activation in the conditions with KCN-blocking oxygen consumption is the sufficient level of ATP previously accumulated in spermatozoa. Subsequently, the motility of the diluted sperm samples studied declined rapidly over time due to depletion of ATP required for sperm movement and an inability to replenish ATP as KCN inhibits oxidative phosphorylation (Nůsková et al. 2010; Pacelli et al. 2011; Perchec et al. 1995).

Thus, both uncoupling of oxidative phosphorylation and inhibition of the respiratory activity of sperm reduce sperm motility over time, but in different ways. Spermatozoa, at the studied dilution rates, preincubated with CCCP, can maintain motility for some time, in contrast to sperm preincubated with KCN. The different patterns of motility reduction may be due to differences in progressive sperm velocity parameters (VSL and LIN), which were lower in sperm preincubated with the respiration inhibitor.

Our study showed that when measuring oxygen consumption using SDR, essential factors affecting oxygen consumption are sperm dilution rate, supplementation, and duration of the experiment, which affect sperm motility percentage and velocity. Therefore, when measuring sperm OCR, it is necessary to work with a specific sperm dilution, considering the possible increase in OCR with an increasing dilution rate. Supplements and time points during measurements should also be selected based on their particular effect on changes in sperm oxygen consumption. The interchangeability of fluorometric and polarographic instruments, as well as the possibility of using an optochemical oxygen sensor to measure oxygen consumption by sperm, provides wide possibilities for combining them for scientific research, considering the concentration of fish sperm, its metabolic state, the need for additives, and the required duration of the experiment. The described respirometry methods complement each other, presenting the potential for studying the respiratory function of sperm of different fish species and opening new perspectives in understanding the diversity of bioenergetic strategies underlying sperm functionality.

## Data Availability

The data presented in this study are available on request from the corresponding author.
